# Determination of HIV status and identification of incident HIV infections in a large, community‐randomized trial: HPTN 071 (PopART)

**DOI:** 10.1002/jia2.25452

**Published:** 2020-02-18

**Authors:** Susan H Eshleman, Estelle Piwowar‐Manning, Ethan A Wilson, Denni Lennon, Jessica M Fogel, Yaw Agyei, Philip A Sullivan, Lei Weng, Ayana Moore, Oliver Laeyendecker, Barry Kosloff, Justin Bwalya, Gerald Maarman, Anneen van Deventer, Sian Floyd, Peter Bock, Helen Ayles, Sarah Fidler, Richard Hayes, Deborah Donnell, for the HPTN 071 (PopART) Study Team

**Affiliations:** ^1^ Department of Pathology Johns Hopkins University School of Medicine Baltimore MD USA; ^2^ Fred Hutchinson Cancer Research Center Seattle WA USA; ^3^ FHI360 Durham NC USA; ^4^ Department of Medicine Johns Hopkins University School of Medicine Baltimore MD USA; ^5^ Zambart University of Zambia School of Medicine Lusaka Zambia; ^6^ Clinical Research Department London School of Hygiene and Tropical Medicine London UK; ^7^ Desmond Tutu TB Center Department of Paediatrics and Child Health Stellenbosch University Stellenbosch Western Cape South Africa; ^8^ Stellenbosch University Stellenbosch Western Cape South Africa; ^9^ Department of Infectious Disease Epidemiology London School of Hygiene and Tropical Medicine London UK; ^10^ Imperial College London London UK

**Keywords:** HIV incidence, seroconverters, HIV testing, community‐randomized, Zambia, South Africa

## Abstract

**Introduction:**

The HPTN 071 (PopART) trial evaluated the impact of an HIV combination prevention package that included “universal testing and treatment” on HIV incidence in 21 communities in Zambia and South Africa during 2013‐2018. The primary study endpoint was based on the results of laboratory‐based HIV testing for> 48,000 participants who were followed for up to three years. This report evaluated the performance of HIV assays and algorithms used to determine HIV status and identify incident HIV infections in HPTN 071, and assessed the impact of errors on HIV incidence estimates.

**Methods:**

HIV status was determined using a streamlined, algorithmic approach. A single HIV screening test was performed at centralized laboratories in Zambia and South Africa (all participants, all visits). Additional testing was performed at the HPTN Laboratory Center using antigen/antibody screening tests, a discriminatory test and an HIV RNA test. This testing was performed to investigate cases with discordant test results and confirm incident HIV infections.

**Results:**

HIV testing identified 978 seroconverter cases. This included 28 cases where the participant had acute HIV infection at the first HIV‐positive visit. Investigations of cases with discordant test results identified cases where there was a participant or sample error (mixups). Seroreverter cases (errors where status changed from HIV infected to HIV uninfected, 0.4% of all cases) were excluded from the primary endpoint analysis. Statistical analysis demonstrated that exclusion of those cases improved the accuracy of HIV incidence estimates.

**Conclusions:**

This report demonstrates that the streamlined, algorithmic approach effectively identified HIV infections in this large cluster‐randomized trial. Longitudinal HIV testing (all participants, all visits) and quality control testing provided useful data on the frequency of errors and provided more accurate data for HIV incidence estimates.

## 1 Introduction

Universal testing and treatment (UTT) for HIV prevention is an important component of HIV prevention programmes 1, 2. The HIV Prevention Trials Network (HPTN) 071 (PopART) trial, the largest HIV prevention trial performed to date, investigated whether UTT and other known effective prevention strategies could reduce HIV incidence on a population level 3. HPTN 071 (PopART) was conducted in 21 urban and peri‐urban communities in South Africa and Zambia. The study included two intervention arms (Arms A and B) and a standard‐of‐care arm (Arm C). Arms A and B included annual home visits with HIV counselling, HIV rapid testing and support for HIV‐infected individuals, including linkage to HIV care and antiretroviral treatment (ART), support for ART adherence and other prevention services 4. ART was initiated at the community health centre at any CD4 cell count (Arm A), or according to local guidelines (Arm B). The impact of the study interventions was measured in a randomly sampled Population Cohort (PC). The PC enrolled> 48,000 adults aged 18 to 44 years; 71% were women. Participants were followed for up to three years. The primary study endpoint was HIV incidence after the first intervention year. HIV incidence was reduced by 30% in communities where ART was provided according to the local guidelines (Arm B vs. C), but was not significantly reduced in communities with UTT (Arm A vs. C) 3.

HIV incidence determination in community‐randomized trials presents unique challenges because of the large number of participants and samples needed for study assessments. To address these challenges, customized approaches were used for sample and data management, HIV testing and determination of HIV status. This report describes the methods that were used to identify incident HIV infections in the trial, and the results obtained. This included identification and characterization of acute and seropositive incident infections and analysis of the performance of HIV screening assays included in the testing algorithms. HIV incidence assessments can be distorted by errors in participant and sample identification (mixups). Determination of HIV status for all PC participants at all visits allowed us to estimate the frequency of those errors and assess the potential impact of those errors on the accuracy of study results.

## 2 Methods

### 2.1 Sample collection, processing and shipping

Samples and data were obtained from PC participants at baseline (PC0) and annual follow‐up visits (PC12, PC24 and PC36) (2013‐2018). At each visit, participants were offered HIV rapid testing, and a 10‐mL blood sample was collected for laboratory‐based HIV testing. This report includes results from only laboratory‐based testing; performance of point‐of‐care HIV rapid testing are reported elsewhere 5. The Laboratory Data Management System was used to track samples throughout the study. Plasma samples were frozen at −80°C within 8 hours of collection. In South Africa, blood samples were processed at a centralized laboratory (SUN Immunology Laboratory; Cape Town) and were tested at the NHLS Laboratory (Tygerberg Hospital, Cape Town). In Zambia, blood samples were processed at one of the five regional laboratories or a central laboratory (Zambart Central Laboratory; Lusaka) and were tested at the Zambart Central Laboratory. Plasma aliquots from all visits were shipped from the in‐country central laboratories to the HPTN Laboratory Center (LC, Johns Hopkins University, Baltimore, MD, USA). A subset of the samples was tested at the LC using pre‐specified testing algorithms.

### 2.2 Laboratory assays

Five assays were used to determine HIV status (Supplemental File 1). Testing was performed at in‐country laboratories using a 4th‐generation HIV test (the CE marked ARCHITECT HIV Ag/Ab COMBO test [Architect]). The LC performed the Architect test (cleared by the United States FDA); a second 4th‐generation test (the GS HIV Combo Ag/Ab EIA [BioRad], performed during PC0, PC12, and PC24); a 5th‐generation test (the BioPlex 2200 HIV Ag/Ab assay [BioPlex], performed during PC36); a discriminatory HIV test (Geenius HIV‐1/2 Supplemental assay [Geenius]); and an HIV RNA test (Abbott RealTime HIV‐1 Viral Load assay [HIV RNA]; validated dilution method, limit of quantification: 400 copies/mL). Selected samples were tested with both the BioRad and BioPlex assays. To streamline testing, samples were tested once with the Architect test at all three laboratories.

### 2.3 Quality control testing

**Figure 1:**
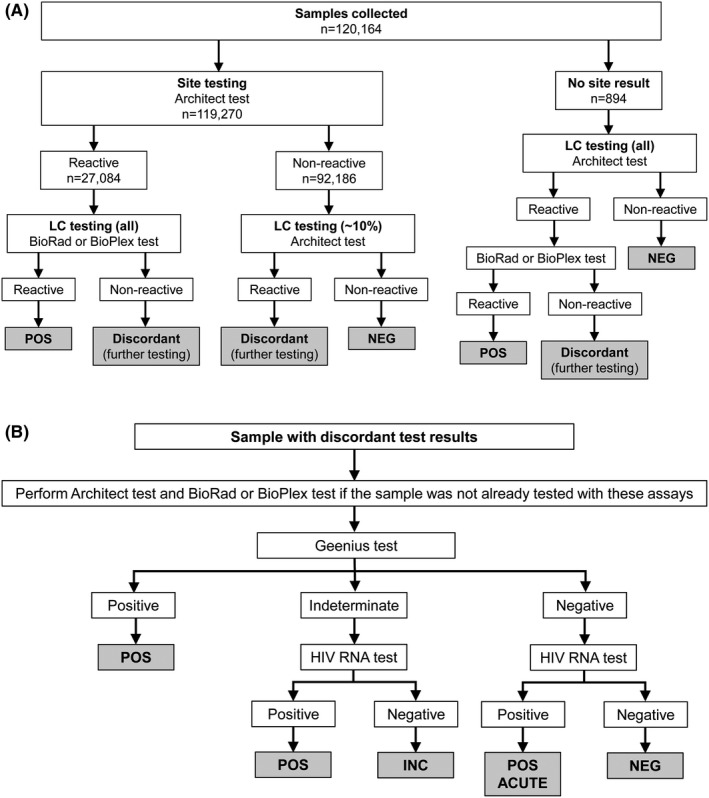
Determination of within‐visit HIV status. Within‐visit HIV status was classified as NEG (HIV uninfected), POS (HIV infectesssd), or INC (inconclusive; HIV status not determined). Results of in‐country testing and quality control (QC) testing at the HPTN Laboratory Center (LC) were compared to identify samples with discordant test results (reactive/non‐reactive). **(A)** shows the QC testing algorithm and **(B)** shows the testing algorithm for samples with discordant site/LC test results. HIV assays: ARCHITECT HIV Ag/Ab COMBO Test (Architect test), GS HIV Combo Ag/Ab EIA (BioRad test), BioPlex 2200 HIV Ag‐Ab Assay (BioPlex test), Geenius HIV‐1/2 Supplemental Assay (Geenius test), and Abbott RealTime HIV‐1 Viral Load Assay (HIV RNA test).

Architect test results from in‐country and LC testing were electronically transferred to the statistical and data management center (SDMC), and laboratory personnel were blinded to study arm throughout the trial. All samples with a reactive in‐country Architect test result were tested at the LC with the BioRad test (PC0, PC12 and PC24) or the BioPlex test (PC36); a random subset (~10%) of the samples with a non‐reactive in‐country Architect test result were tested at the LC using the same assay (Architect test) (Figure 1).

### 2.4 Determination of within‐visit HIV status

The results of in‐country testing and QC testing were compared to identify samples with discordant results (reactive/non‐reactive). Additional testing was performed at the LC for those samples (Figure 1). Within‐visit HIV status was classified as NEG (HIV uninfected), POS (HIV infected), or INC (inconclusive; Supplemental File 2).

### 2.5 Determination of across‐visit HIV status

**Figure 2:**
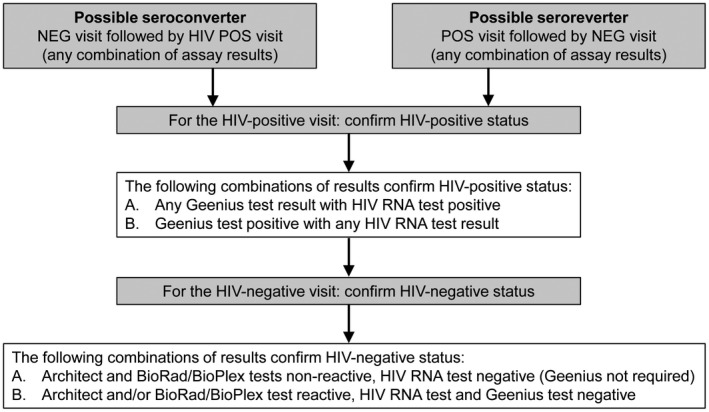
Confirmation of seroconverter and seroreverter cases. The figure shows the testing strategy used to confirm seroconverter and seroreverter cases. NEG, negative; POS, positive.

HIV test results were compared across study visits to identify samples that required additional testing. This included cases that had a NEG visit followed by a visit with a reactive test (potential seroconverter), cases that had a POS or INC visit followed by a visit with a non‐reactive test (potential “seroreverter,” indicating possible participant/sample mixups) and other cases with discrepant test results. Across‐visit HIV status (HIV status based on the analysis of samples from longitudinal study visits) was determined using in‐country and LC test results. Cases were provisionally classified as HIV POS (HIV infected at all visits), HIV NEG (HIV uninfected at all visits), potential seroconverter, potential seroreverter, or to be determined (across‐visit status unclear due to missing and/or discrepant HIV test results). For seroconverter and seroreverter cases, additional testing was performed at selected study visits to confirm the change in HIV infection status (Figure 2). In confirmed seroconversion cases, samples collected at the last NEG visit were also tested with the HIV RNA test to determine if the participant had acute infection at that visit. Samples were classified as having acute infection (POS ACUTE) if the Geenius test was negative and HIV RNA was detected. Additional visits with acute HIV infection were identified at study entry and at end‐of‐study visits during the evaluation of samples with discordant test results.

### 2.6 Endpoint adjudication

Within‐visit and across‐visit HIV status were determined at the LC (by manual review of test results) and at the SDMC (using computerized algorithms). Cases that had concordant LC and SDMC HIV status determinations were not reviewed further. All the remaining cases were reviewed by a Virology Endpoint Adjudication Committee (VEAC) that included two virologists from the LC and three external virologists (Supplemental File 2).

### 2.7 Evaluation of errors due to participant or sample mixups

We evaluated the frequency of errors due to participant or sample mixups by comparing within‐visit HIV status for participants who had paired HIV status results from consecutive visits in three time intervals: PC0‐PC12, PC12‐PC24 and PC24‐PC36. Statistical methods used to derive error rates and the probability of true incident cases are shown in Supplemental File 3. Briefly, for this analysis, the error rate, *m*, represents probability that test results from a visit do not belong to the designated study participant. The error rate was evaluated by determining the proportion of cases where the within‐visit HIV status changed from POS to NEG (${\hat{p}}_{\mathrm{PN}})$, where ${\hat{p}}_{1}and{\hat{p}}_{2}$ represent the observed prevalence of a POS within‐visit status at the first or second of the paired visits respectively:

$$m=1-\sqrt{1-\frac{{\hat{p}}_{\mathrm{PN}}}{{\hat{p}}_{1}\left(1-{\hat{p}}_{2}\right)}}$$

After accounting for the probability of errors, the probability, $\hat{d}$, of a true incident infection among those who were HIV uninfected at the first visit where both samples came from the same study participant is as follows:

$$\hat{d}=\frac{{\hat{p}}_{2}-{\hat{p}}_{1}}{\left(1-{\hat{p}}_{1}\right)}$$

### 2.8 Informed consent

Written informed consent was obtained from all PC participants 3. Ethical approval for the trial was provided by committees at London School of Hygiene and Tropical Medicine, University of Zambia, and Stellenbosch University.

## 3 Results

### 3.1 Samples used for analysis

Overall, 48,301 participants were enrolled in the PC. HIV status was evaluated in 47,470 PC participants; 831 (1.7%) participants had no samples available for testing (missing HIV status). Table 1 shows the number of samples tested for each enrolment group in each survey year (total 120,164 samples tested). Overall, 278 (0.23%) of the 120,154 samples had discordant test results; there was no significant difference in the frequency of discordant test results by age, sex, study country or study arm (data not shown).

**Table 1:** Number of samples analysed by enrolment cohort and survey year

Enrolment	Annual survey				Total
group	PC0	PC12	PC24	PC36	
PC0	**37,320**	22,925	20,028	20,029	100,302
PC12N	‐‐	**4,844**	3,415	3,362	11,621
PC24N	‐‐	‐‐	**4,650**	3,591	8,241
TOTAL	37,320	27,769	28,093	26,982	120,164

Participants were enrolled in the HPTN 071 Population Cohort (PC) during a baseline survey (PC0) and during annual surveys conducted one or two years later (PC12N and PC24N respectively; total enrolled: 48,301). Samples were collected at baseline (PC0) and during three annual surveys (PC12, PC24 and PC36) from participants who consented to sample collection (total participants with at least one sample available: 47,470); 831 cases had no sample available from any study visit (e.g. no study visit, participant refused sample collection, unsuccessful blood draw). The table shows the number of samples analysed from each of the four annual surveys (PC0, PC12, PC24 and PC36) from each enrolment group (PC0, PC12N and PC24N). Samples from enrolment visits are shown in bold text (total number of enrolment samples: 46,814).

### 3.2 Determination of within‐visit HIV status for samples that had a non‐reactive or missing in‐country test result

Architect test results were obtained in‐country for 119,270 (99.3%) of the 120,164 samples collected. The remaining 894 samples had no aliquot available for in‐country testing or did not have a test result. The in‐country Architect test was non‐reactive for 92,186 (77.3%) of the 119,270 samples tested. The same assay was performed at the LC for 10,731 (11.6%) of the 92,186 samples; this included samples that were randomly selected for QC testing (Figure 1A) and samples that had additional testing performed at the LC to determine across‐visit HIV status. The LC Architect test was non‐reactive for 10,680 (99.5%) of the 10,731 samples, confirming the results of the in‐country test. Those samples were classified as NEG and were not analysed further unless the participant had a change in HIV status across visits.

In the remaining 51 cases (0.5%), results of the two Architect tests were discordant (non‐reactive in‐country test, reactive LC test). Those samples were tested at the LC with the BioRad or BioPlex test, the Geenius test and the HIV RNA test (Supplemental File 4A). HIV status was not determined for one sample (classified as INC). Further testing of the remaining 50 samples confirmed that 27 were HIV negative and 23 were HIV positive. The discrepancy between S/CO values obtained at the site and LC suggest that the majority of these were sample or data mixups (Supplemental File 4B). The analysis also indicated that triplicate testing with the Architect test for samples with S/CO ratios ≥ 1 (as recommended by the manufacturer) would not have resolved most of the discrepancies (Supplemental File 4C).

### 3.3 Determination of within‐visit HIV status for samples that had a reactive in‐country test result

Overall, 27,084 (22.7%) of the 119,270 samples had a reactive in‐country Architect test; 26,972 (99.6%) of those samples were tested with a second HIV screening test at the LC (BioRad or BioPlex test; Figure 1). A reactive BioRad or BioPlex test was obtained for 26,745 (99.2%) of the samples. The remaining 227 samples (0.8%) had discordant test results (reactive in‐country Architect test, non‐reactive LC BioRad or BioPlex test); those samples were tested at the LC with the Geenius test and HIV RNA test (Figure 1B). Overall, 206 (90.7%) of the 227 samples were confirmed to be from HIV‐uninfected individuals (Supplemental File 5A). The remaining 21 samples included eight that were confirmed to be HIV positive (including three acute samples) and 13 with inconclusive HIV status. Additional testing was performed to evaluate the reasons for the discordant test results. That analysis indicated that some discordant results reflected laboratory errors (sample or data mixups; testing or data errors), while others were likely explained by assay variability (Supplemental File 5B).

Sixty additional samples had reactive results with the LC Architect test and the LC BioRad or BioPlex test, but were confirmed to be from HIV‐uninfected individuals (data not shown). Those cases, which had false reactive results with two different screening tests, were identified during evaluation of possible seroconverter or seroreverter cases.

### 3.4 Determination of across‐visit HIV status

Across‐visit HIV status was provisionally determined for each participant by analysing test results from longitudinal study visits; a pre‐determined plan was used to identify cases that required additional adjudication to determine across‐visit HIV status, to determine the timing of seroconversion events, and to identify participants who had an acute HIV infection visit (see Methods). Overall, 369 (0.76%) of the 48,301 cases were referred for VEAC review. The final across‐visit status changed in 69 (28.7%) of those cases and two cases that were not referred for review (Supplemental File 2).

### 3.5 Identification of seroconverter cases

After accounting for the 831 cases with missing HIV status, 16 ND cases, 213 seroreverter cases and 10,051 cases where the participant was HIV infected at enrolment, 37,190 cases remained where the participant was HIV uninfected at enrolment; 26,498 (71.3%) of these cases had at least one sample tested from a subsequent study visit. Potential seroconverter events were identified when a visit classified as NEG was followed by a visit where the in‐country test result or the LC QC test result was reactive; additional testing was performed at the LC in these cases (Figures 1B and 2). All potential seroconverters were further classified based on the timing of the last NEG and first POS visits (e.g. SC0‐12, for the last NEG visit at PC0 and the first POS visit at PC12). Overall, 978 seroconverter cases were identified after adjudication (Table 2); 752 (77%) of the seroconverters had detectable HIV RNA at the first POS visit (two did not have a viral load test at this visit). The median HIV viral load at the first POS visit in these 752 cases was 14,435 copies/mL (range: 400 to> 16 million). The percentage of seroconverter cases with viral loads < 400 copies/mL increased over time (25% at PC12, 30% at PC24, 33% at PC36; 29% overall, all three visits).

**Table 2:** Final across‐visit HIV status

HIV status	Number of cases	Percentage of cases
Across‐visit HIV status		
Missing	831	1.72
Negative all visits	36,212	74.97
Positive all visits	10,051	20.81
Seroreverter^a^	213	0.44
Not determined	16	0.03
Seroconverter	978	2.03
Total	48,301	
Seroconverter type		
Primary endpoint NEG at PC12^b^	505	
Primary endpoint – no status at PC12^b^	48	
Acute infection at first POS visit^c^	28	

Seroconverter timing	Number of cases	Percentage of seroconverters
PC0‐12	360	36.81
PC0‐24	67^b^	6.85
PC0‐36	46^b^	4.70
PC12‐24	225	23.01
PC12‐36	57	5.83
PC24‐36	223	22.80
TOTAL	**978**	

The table shows the final across‐visit HIV status for 48,301 participants enrolled in the HPTN 071 Population Cohort. Of these, 831 had no samples available for analysis (missing) and 16 did not have HIV status determined due to missing and/or inconclusive HIV test results. HIV status was determined on at least one visit for the remaining 47,454 participants. Classifications included: HIV NEG (HIV negative at all visits with an HIV status); HIV POS (HIV positive at all visits with an HIV status; in some of these cases, the participant had acute HIV infection at the first HIV‐positive visit); seroreverter (confirmed HIV‐positive visit followed by a confirmed HIV‐negative visit); seroconverter (confirmed HIV‐negative visit followed by a confirmed HIV‐positive visit) (see Supplemental Table 2).

a In all 213 seroreverter cases, participants had a positive Geenius test before testing negative for HIV infection; there were no seroreversion cases where the only HIV‐positive visit was a visit with acute HIV infection. The overall rate of seroreversion events among the subset of cases with HIV status determined at two or more visits was 0.64%. Seroreversion was not associated with sex or study arm; a slightly higher frequency of seroreversion cases was observed in Zambia compared to South Africa (0.75% vs. 0.47%), and among older participants (0.78% in those age > 24 vs. 0.41% in those ages 18 to 24)”

b Seroconverters who were HIV uninfected at the PC12 visit were included in the primary study endpoint analysis. This included 225 cases classified as SC12‐24, 223 cases classified as SC24‐36, and 57 cases classified as SC12‐36. In 113 cases classified as SC0‐24 or SC0‐36, participants did not have HIV status determined at PC12 due to a missed study visit or no sample collected; 48 of these cases contributed to the primary endpoint using statistical imputation methods (see Methods)

c Participants who had acute HIV infection at enrolment were not classified as seroconverters. Participants who were HIV NEG at PC0 and had acute HIV infection at PC12 were classified as seroconverters, but were not included in the primary endpoint analysis.

### 3.6 Evaluation of cases with acute HIV infection

Twenty‐eight cases of acute infection were identified (Table 2). Twenty‐two of these cases had a subsequent visit with documented HIV seroconversion (reactive Geenius test); in the other six cases, acute infection was detected at the last study visit. A detailed description of these cases is presented in Supplemental File 6.

### 3.7 Evaluation of the potential impact of sample/data mixups on HIV incidence estimation

Overall, 33,408 cases had HIV test results from at least two study visits; 213 (0.64%) of those cases had results that indicated an error or specimen mixup (seroreverter cases). In 154 of the 213 cases, the participant had a POS status at enrolment; those cases were never considered eligible for the incidence analysis. In the remaining 59 cases, the participant had a NEG status at enrolment, and had a sample from a subsequent visit with confirmed HIV infection (Supplemental File 7). Those 59 cases were provisionally classified as seroconversion events; however, the classification was later changed from seroconverter to seroreverter, because a sample from a later study visit was confirmed to be HIV negative. Those 59 cases included 33 cases where the participant had a NEG status at PC12; those 33 cases would have been included as primary endpoint events if the seroreversion event was not detected.

While seroreverter cases provided clear evidence of sample/participant mixups, this type of error could have occurred in other cases without being detected, and could have led to misclassification of seroconverter cases (e.g. if the sample used to determine HIV status at the first POS visit was from a different participant and the participant had no subsequent study visit). We used data from the seroreversion cases to estimate the impact of these errors on the accuracy of identification of incident infections (Table 3, Supplemental File 3). In this analysis, data from paired sequential study visits were analysed in three different time intervals. We identified 47 seroreversion events (errors) in the interval PC0‐12 (0.21%), 64 seroreversion events in the interval PC12‐24 (0.31%) and 75 seroreversion events in the interval PC24‐36 (0.32%). In the first interval (PC0‐12), there were 384 apparent incident cases (i.e. cases where within‐visit HIV status changed from NEG to POS; 2.20% of cases analysed); 24 of those cases were classified as seroreverter cases, because the participant had a NEG within‐visit HIV status at a subsequent visit. After removing those 24 cases, 360 of the 384 cases remained classified as seroconverters (observed incident cases; 2.03% of cases analysed). Using the observed rate of mixups to estimate the overall (unobserved) error rate, the estimated frequency of true incident infections in this time interval was 1.90% (corrected incidence rate). The same approach was used to calculate the observed (uncorrected) and corrected number of incident cases in each time interval. In each of the three time intervals, exclusion of seroreversion cases removed at least half of the potential seroconverter cases that were likely to represent participant or sample errors (Table 3).

**Table 3:** Analysis of within‐visit HIV status for paired visits to assess the impact of participant/sample mixups on reported HIV incidence

	First year of follow‐up		Second year of follow‐up		Third year of follow‐up	
Observed data pairs^a^	PC0, PC12 (N = 22,555)	%	PC12, PC24 (N = 20,693)	%	PC24, PC36 (N = 23,802)	%
Negative (N→N)	17,401	77.15%	15,866	76.67%	18,159	76.29%
Positive (P→P)	4,723	20.94%	4,489	21.69%	5,305	22.29%
Possible seroconverter (N→P)	384	1.70%	274	1.32%	263	1.10%
Seroreverters (errors) (P→N)	47	0.21%	64	0.31%	75	0.32%
Estimated error rate (m^b^)		0.64%		0.92%		0.91%
Proportion infected, first visit (p1)		21.15%		22.00%		22.60%
Proportion infected, second visit (p2)		22.64%		23.02%		23.39%
**Expected number of errors** (based on error rate^c^)						
Seroconverter (N→P)	51		68		78	
Negative (N→N)	175		227		257	
**Observed number of errors** (cases removed following adjudication)						
Seroconverter (N→P)	24		49		40	
Negative (N→N)	26		21		33	
**Observed (uncorrected) proportion incident** d	384/ (17,401 + 384)	2.20%	274/ (15,866 + 274)	1.72%	263/ (18,159 + 263)	1.44%
**Reported proportion incident** e (removing observed errors)	(384‐24)/ (17,401‐26 + 384‐24)	2.03%	(274‐49)/ (15,866‐21 + 274‐49)	1.40%	(263‐40)/ (18,159‐33 + 263‐40)	1.22%
**Probable true proportion incident** f (based on paired final HIV results)	(384‐51)/ (17,401‐175 + 384‐51)	1.90%	(274‐68)/ (15,866‐227 + 274‐68)	1.30%	(263‐78)/ (18,159‐257 + 263‐78)	1.02%

a Data indicate the number of participants with samples collected at two consecutive study visits (PC0 and PC12; PC12 and PC24; PC24 and PC36). N→N: participants classified as HIV NEG at both visits; P→P: participants classified as HIV POS at both visits; N→P: participants classified as HIV NEG at the first visit and HIV POS at the subsequent visit; P→N: participants classified as HIV POS at the first visit and HIV NEG at the subsequent visit (these cases represent observed errors in participant or sample identification at one or both study visits)

b The symbol, m, represents the estimated error rate (the estimated proportion of cases where within‐visit HIV status was incorrect at one or both visits due to a participant or sample mixup)

c These estimates are based on the estimated error rate (m)

d This shows the proportion of incident cases and incidence rate observed without excluding seroreverter cases (observed errors)

e This shows the proportion of incident cases and incidence rate after excluding seroreverter cases (observed errors)

f This shows the probable (true) proportion of incident cases and incidence rate based on analysis of paired within‐visit HIV status results, adjusting for unobserved errors due to participant or sample mixups.

## 4 DISCUSSION

This report describes the methods used to determine HIV status and identify incident infections in the HPTN 071 (PopART) trial. The size of this trial (48,301 participants followed for up to three years; >120,000 samples tested) presented challenges in sample and data management. A streamlined approach was used to reduce the cost, effort and complexity of HIV testing. Customized data management procedures were used to reduce the frequency of clerical errors. An external adjudication committee reviewed > 300 cases with complex test results. Across‐visit HIV status was determined in all but 16 cases. Limitation of testing for many samples to a single HIV screening test had a minimal impact on study results.

In a previous community‐randomized study, the primary HIV incidence endpoint was determined by analysing samples collected in a cross‐sectional survey of > 46,000 individuals 6. In that study, the testing algorithm used for cross‐sectional HIV incidence estimation included viral load as a biomarker for non‐recent infection 7; low viral load is also used as a biomarker for non‐recent infection in an algorithm that is widely used for cross‐sectional HIV incidence estimation in surveillance studies 8, 9. Further studies are needed to assess the performance of these algorithms in settings where ART is initiated early in HIV infection since individuals with recent infection who are virally suppressed from ART would be misclassified as having non‐recent infection.

Overall, 978 seroconverter cases were identified; 553 of these cases were used for the primary HIV incidence analysis 3. The viral loads were < 400 copies/mL in 29% of all seroconverter cases. In these cases, HIV infection may have been diagnosed in the community between study visits and the participant may have initiated ART before the seroconversion was documented by study testing; in some cases, the participant may have been virally suppressed in the absence of ART. The frequency of low viral load seroconverter samples increased over time, suggesting an increase in earlier diagnoses and ART initiation over the course of the study.

Twenty‐eight participants had acute HIV infection at the first HIV‐positive visit. In four cases, the viral load was <400 copies/mL at the acute visit. Since the 978 seroconversion events were likely distributed evenly over the year preceding the first HIV‐positive visit, we would expect that some seroconverter events would be detected during the acute infection period (e.g. within one week of HIV infection) and that a portion of those cases would be detected only 1‐2 days after infection, at a time when viral load might be low. The sensitivity for detecting acute infections was similar for the three laboratory‐based antigen/antibody HIV screening tests (Architect, BioRad, BioPlex). The 5th‐generation test (BioPlex) identified only 25% of the acute samples as positive for HIV antigen only. ART initiation during acute infection can suppress viraemia and HIV antibody expression; loss of HIV antibodies (seroreversion) has been described in some cases 10. In HPTN 071, it is unlikely that participants would have initiated ART during the acute phase of HIV infection since HIV testing offered at home visits was performed using 3rd‐generation HIV rapid tests. Furthermore, the laboratory‐based testing described in this report did not identify any seroreversion cases where an acute infection visit was followed by a confirmed HIV‐negative visit.

This report included comparison of Architect test results obtained in‐country and at the HPTN LC for >10,000 samples. Two non‐reactive results were obtained in 10,680 (99.5%) of these cases. We evaluated cases with discordant Architect tests, and cases where the in‐country Architect test and LC BioRad/BioPlex tests were discordant. Investigation indicated that some of these cases likely represented laboratory errors (aliquot mixups, or sample/data errors). Other cases involving participant or sample mixups were identified by longitudinal testing (seroreverter cases). Longitudinal HIV testing is not performed in most studies once an individual is determined to be HIV infected 11; therefore, most studies will not detect these errors. These cases were relatively rare in HPTN 071 (PopART), considering the size of the study (0.4% of 47,470 cases evaluated). Seroreverter cases were excluded from the analysis of HIV incidence. Using statistical methods, the frequency of seroreverter cases was used to estimate the possible number of undisclosed mixups that may have resulted in incorrect identification of seroconverter events or failure to identify seroconverter events. Statistical analysis showed that exclusion of seroreverter cases from HIV incidence analysis improved the accuracy of the incidence estimate.

## 5 Conclusions

This report demonstrates that the streamlined, algorithmic approach was effective for identifying incident HIV infections in this large community‐randomized trial. The report also demonstrates the utility of QC testing for investigation of discordant test results, the value of performing HIV testing for all participants at all visits, removal of seroreverter cases and the use of statistical methods for investigating the impact of sample mixups on HIV incidence estimates increased the accuracy of HIV incidence estimates.

## Competing interests

None of the authors has a conflict of interest or potential conflict of interest, with the following exceptions: Susan Eshleman has collaborated on research studies with investigators from Abbott Diagnostics; Abbott Diagnostics provided reagents for previous research studies.

## Authors’ contributions

All authors have read and approved the final manuscript. Additional author roles are listed below.

**Table:** 

Susan H. Eshleman	HPTN 071 Protocol Virologist; designed the laboratory plan for HPTN 071; conceived of the study, analysed data, drafted the manuscript
Estelle Piwowar‐Manning	HPTN 071 QAQC Coordinator; designed the laboratory plan and coordinated laboratory testing for HPTN 071; responsible for HIV testing at the HPTN LC; analysed data
Ethan A. Wilson	HPTN 071 Statistical Research Associate
Denni Lennon	Coordinated HIV testing at the HPTN LC; analysed data
Jessica M. Fogel	Analysed data; assisted with manuscript writing
Yaw Agyei	HPTN 071 International QAQC Coordinator; assisted with coordination of laboratory testing for HPTN 071
Philip Sullivan	Assisted with testing and review of HPTN 071 laboratory data
Lei Weng	HPTN 071 Lab Data Coordinator at SDMC
Ayana Moore	HPTN 071 Study Coordinator
Oliver Laeyendecker	Assisted with presentation of HPTN 071 laboratory data
Barry Kosloff	HPTN 071 Laboratory Manager in Zambia
Justin Bwalya	HPTN 071 Population Cohort Coordinator in Zambia
Gerald Maarman	HPTN 071 Research Manager in South Africa
Anneen van Deventer	HPTN 071 Laboratory Coordinator in South Africa
Sian Floyd	HPTN 071 Senior Statistician (LSHTM)
Peter Bock	HPTN 071 South African Site Co‐PI
Helen Ayles	HPTN 071 Zambian Site PI
Sarah Fidler	HPTN 071 Protocol Co‐Chair
Richard Hayes	HPTN 071 Protocol Chair
Deborah Donnell	HPTN 071 Protocol Statistician

## Supporting information


**File 1.** Assays used to determine HIV status.
**File 2.** Determination of within‐visit and across‐visit HIV status.
**File 3**. Derivation of estimates for error rate and probability of true incident cases for paired samples from sequential visits.
**File 4.** Analysis of 51 samples that had a non‐reactive in‐country Architect test and a reactive HPTN LC Architect test.
**File 5.** Analysis of 227 samples that had a reactive in‐country Architect test and a non‐reactive HPTN LC BioRad or BioPlex test.
**File 6.** Characteristics of acute HIV infections.
**File 7.** Pattern of HIV test results in seroreverter cases.
