# Supplementary material for: Determination of HIV status and identification of incident HIV infections in a large, community‐randomized trial: HPTN 071 (PopART)
Source: J Int AIDS Soc. 2020 Feb 18;23(2):e25452. doi: 10.1002/jia2.25452 (PMC7028526; doi:10.1002/jia2.25452)
Supplement: Supplementary file 1 — File 1. Assays used to determine HIV status. File 2. Determination of within‐visit and across‐visit HIV status. File 3. Derivation of estimates for error rate and probability of true incident cases for paired samples from sequential visits. File 4. Analysis of 51 samples that had a non‐reactive in‐country Architect test and a reactive HPTN LC Architect test. File 5. Analysis of 227 samples that had a reactive in‐country Architect test and a non‐reactive HPTN LC BioRad or BioPlex test. File 6. Characteristics of acute HIV infections. File 7. Pattern of HIV test results in seroreverter cases. [file JIA2-23-e25452-s001.docx]

**SUPPLEMENTAL FILES**

Supplemental File 1. Assays used to determine HIV status.

| **Assay name** | **Manufacturer** | **Short name** | **Assay type** | **Analytes** | **Results used to determine HIV status** |
| --- | --- | --- | --- | --- | --- |
| ARCHITECT HIV Ag/Ab COMBO Test | Abbott Diagnostics, Wiesbaden, Germany | Architect test | 4^th^-generation chemiluminescent microparticle immunoassay | HIV-1 Ag, HIV-1 Ab, HIV-2 Ab (reported as a single result) | Reactive  Non-reactive |
| GS HIV Combo Ag/Ab EIA | Bio-Rad Laboratories, Hercules, CA | BioRad test | 4^th^-generation EIA | HIV-1 Ag, HIV-1 Ab, HIV-2 Ab (reported as a single result) | Reactive  Non-reactive |
| BioPlex 2200 HIV Ag-Ab Assay | Bio-Rad Laboratories, Hercules, CA | BioPlex test | 5^th^-generation multiplex flow assay | (HIV-1 Ag, HIV-1 Ab, HIV-2 Ab (reported as separate results) | Reactive  Non-reactive |
| Abbott RealTime HIV-1 Viral Load Assay | Abbott Molecular, Des Plaines, IL | HIV RNA test | HIV RNA quantification, validated dilution method | HIV RNA; limit of quantification: 400 copies/mL HIV RNA | Detected^a^  Not detected |
| Geenius HIV-1/2 Supplemental Assay | Bio-Rad Laboratories, Hercules, CA | Geenius test | Immunochromatographic differentiation test | HIV-1 Ag, HIV-1 Ab, HIV-2 Ab,  (reported as separate results, plus an overall result) | Positive  Negative  Indeterminate |

Footnotes for Supplemental File 1.

^a^ Includes samples with HIV RNA detected below the limit of quantification.

Abbreviations: HIV-1: Human immunodeficiency virus type 1; HIV-2: human immunodeficiency virus type 2; Ag: antigen; Ab: antibody; EIA: enzyme immunoassay.

Supplemental File 2. Determination of within-visit and across-visit HIV status.

Within-visit and across-visit HIV status were determined separately by analyzing data at the HPTN LC and SDMC (see Table below). Cases with uncomplicated test results that had concordant LC and SDMC HIV status determinations were not reviewed further. This included cases where the within-visit status was NEG at all visits; cases where the within-visit status was POS at all visits; and uncomplicated seroconverter cases (where the within-visit HIV status was NEG for all visits prior to the first POS visit, the within-visit status was POS for all visits after the first POS visit, and there was no visit with acute HIV infection). The Virology Endpoint Adjudication Committee (VEAC) reviewed all remaining cases; this committee included five virologists with expertise in HIV diagnosis.

The HPTN SDMC compiled data for cases that required adjudication and posted the data to a secure web site. Cases were reviewed independently online by each virologist. Final across-visit classification options included HIV POS, HIV NEG, seroconverter, seroreverter, and ND (HIV status could not be determined based on available data). For SC cases, the virologists entered the following information in additional data fields: timing of the last NEG visit, timing of the first POS visit, acute infection at the first POS visit (true/false); and the timing of the acute infection visit, if an acute infection visit was identified (these visits were classified as the first POS visit).

Adjudication decisions were considered final if four of the five reviewers entered the same classification for HIV status, and entered the same determinations in all fields for seroconversion cases. If the entries were not concordant among at least four of the five reviewers, or if any of the reviewers flagged a case for further discussion, the cases were reviewed on conference calls that included the five virologists, the Protocol Statistician, and Statistical Research Associate; the final status of those cases was determined by consensus of the group.

Overall, the final across-visit status changed in 69 (28.7%) of the 369 cases that were referred to the VEAC for review, and in two additional cases that were not referred for VEAC review. The 71 cases where across-visit status was changed included 1 case that changed from POS to ND; two cases that changed from seroreverter to ND; five cases that changed from seroreverter to NEG; and 63 TBD cases, including 35 changed to NEG, 12 changed to ND, eight changed to seroconverter, and eight changed to seroreverter. The results presented below are based on final (post-adjudication) across-visit HIV status. In 16 cases, across-visit HIV status could not be determined because of missing or inconclusive HIV test results; 213 cases had HIV test results that indicated an error or specimen mixup (seroreverter cases). This included 59 cases in which the participant was uninfected at study enrollment.

| **Classification** | **Description** | **Relevance to HIV incidence estimation** |
| --- | --- | --- |
| Within visit (sample) |  |  |
| POS | HIV infected |  |
| POS ACUTE^a^ | Acute HIV infection |  |
| NEG | HIV uninfected |  |
| INC | HIV status inconclusive |  |
| Across visits (participant) | | |
| HIV POS | HIV infected at all visits | Prevalent positive case; not considered in the estimation of HIV incidence |
| HIV POS ACUTE | HIV infected at all visits; acute infection at the first HIV POS visit | Prevalent positive case; not considered in the estimation of HIV incidence |
| HIV NEG | HIV uninfected at all visits | Uninfected (at risk); included in HIV incidence estimation |
| Seroconverter^b^ | HIV seroconverter (NEG🡪POS) | Incident infection (study endpoint); included in HIV incidence estimation |
| Seroreverter | “Seroreverter” (POS🡪NEG) | Data indicate a participant or sample mix-up; HIV status was censored at all visits for these cases; not included in HIV incidence estimation |
| ND | HIV status not determined | Status not determined; not included in HIV incidence estimation |

Table footnotes.

^a^Testing to identify acute infection was performed for a subset of samples based on across-visit test results; the acute visit was considered to be the first HIV-positive visit.

^b^Seroconverter cases that were NEG at PC12 were primary study endpoints; cases that had missing HIV status at PC12 were imputed to infer the timing of the first HIV POS visit (approximately ½ of these cases were primary study endpoints).

Abbreviations: POS: positive; NEG: negative; INC: inconclusive; ND: not determined.

Supplemental File 3. Derivation of estimates for error rate and probability of true incident cases for paired samples from sequential visits.

Notation:

Let $s_{i}\left( v_{j} \right)=0$if the sample stored with studyID $i$ at visit $v_{j}$ comes from participant $i$, 1 otherwise (i.e., there was a participant or sample mixup). Let $T\left( s \right)=0$ if the observed final within-visit HIV status is negative, 1 if positive. For a pair of visits, $v_{1}$ and $v_{2}$let ${p_{1}, p}_{2}$be the probabilities of a positive within-visit status at $v_{1}$ and $v_{2},$respectively. Let *m* be the probability of an error, and assume the probability of an error is the same at both visits, $m = P\left( s_{i}\left( v_{1} \right)=1 \right)=P\left( s_{i}\left( v_{2} \right)=1 \right)$. Let *d* be the probability of a true incident infection (i.e., positive within-visit status at visit at $v_{2}$ amongst those with a negative within-visit HIV status at $v_{1}$ when both samples come from the identified participant), where $d = P\left( T\left( s_{i}\left( v_{2} \right) \right)=1 | {T\left( s_{i}\left( v_{1} \right) \right)=0, s}_{i}\left( v_{1} \right)=0,s_{i}\left( v_{2} \right)=0 \right)$. With a straightforward application of Bayes theorem, we can calculate:

$$p_{2}= P\left( T\left( s_{i}\left( v_{2} \right) \right)=1 \right)=P\left( T\left( s_{i}\left( v_{2} \right) \right)=1,s_{i}\left( v_{2} \right)=1 \right)+P\left( T\left( s_{i}\left( v_{2} \right) \right)=1,s_{i}\left( v_{2} \right)=0 \right)=P\left( T\left( s_{i}\left( v_{2} \right) \right)=1,s_{i}\left( v_{2} \right)=1 \right)+P\left( T\left( s_{i}\left( v_{2} \right) \right)=1,s_{i}\left( v_{2} \right)=0 , s_{i}\left( v_{1} \right)=1 \right)+P\left( T\left( s_{i}\left( v_{2} \right) \right)=1,s_{i}\left( v_{2} \right)=0 , T\left( s_{i}\left( v_{1} \right) \right)=0, s_{i}\left( v_{1} \right)=0 \right)+P\left( T\left( s_{i}\left( v_{2} \right) \right)=1,s_{i}\left( v_{2} \right)=0 , T\left( s_{i}\left( v_{1} \right) \right)=1, s_{i}\left( v_{1} \right)=0 \right)=P\left( s_{i}\left( v_{2} \right)=1 | T\left( s_{i}\left( v_{2} \right) \right)=1 \right)P\left( T\left( s_{i}\left( v_{2} \right) \right)=1 \right)+P\left( T\left( s_{i}\left( v_{2} \right) \right)=1 | s_{i}\left( v_{1} \right)=1,s_{i}\left( v_{2} \right)=0 \right)P\left( s_{i}\left( v_{1} \right)=1,s_{i}\left( v_{2} \right)=0 \right)+P\left( T\left( s_{i}\left( v_{2} \right) \right)=1, | T\left( s_{i}\left( v_{1} \right) \right)=0, s_{i}\left( v_{1} \right)=0,s_{i}\left( v_{2} \right)=0 \right) \times P\left( T\left( s_{i}\left( v_{1} \right) \right)=0 | s_{i}\left( v_{1} \right)=0,s_{i}\left( v_{2} \right)=0 \right)P\left( s_{i}\left( v_{1} \right)=0,s_{i}\left( v_{2} \right)=0 \right)+P\left( T\left( s_{i}\left( v_{2} \right) \right)=1,T\left( s_{i}\left( v_{1} \right) \right)=1 | s_{i}\left( v_{1} \right)=0,s_{i}\left( v_{2} \right)=0 \right)P\left( s_{i}\left( v_{1} \right)=0,s_{i}\left( v_{2} \right)=0 \right)=mp_{2}+\left( 1-m \right)^{2}\left( d\left( 1-p_{1} \right)+p_{1} \right)+m\left( 1-m \right)p_{2}$$

From this we can conclude:

$$d = \frac{p_{2}-p_{1}}{\left( 1-p_{1} \right)}$$

Also, when a negative within-visit status is observed after a positive within-visit status:

$$p_{PN} = P\left( T\left( s_{i}\left( v_{1} \right) \right)=1,T\left( s_{i}\left( v_{2} \right) \right)=0 \right)=P\left( T\left( s_{i}\left( v_{1} \right) \right)=1,s_{i}\left( v_{1} \right)=0,T\left( s\left( v_{2} \right) \right)=0,s_{i}\left( v_{2} \right)=0 \right)+P\left( T\left( s_{i}\left( v_{1} \right) \right)=1,s_{i}\left( v_{1} \right)=0,T\left( s_{i}\left( v_{2} \right) \right)=0,s_{i}\left( v_{2} \right)=1 \right)+P\left( T\left( s_{i}\left( v_{1} \right) \right)=1,s_{i}\left( v_{1} \right)=1,T\left( s_{i}\left( v_{2} \right) \right)=0,s_{i}\left( v_{2} \right)=0 \right)+P\left( T\left( s_{i}\left( v_{1} \right) \right)=1,s_{i}\left( v_{1} \right)=1,T\left( s_{i}\left( v_{2} \right) \right)=0,s_{i}\left( v_{2} \right)=1 \right)=0 + {2m\left( 1-m \right)p}_{1}\left( 1-p_{2} \right) + {m^{2}p}_{1}\left( 1-p_{2} \right) = m{\left( 2-m \right)p}_{1}\left( 1-p_{2} \right)$$

From this we can calculate:

$$m=1-\sqrt{1-\frac{p_{PN}}{p_{1}\left( 1-p_{2} \right)}}$$

Estimates of m and d are computed by replacing ${p_{1}, p}_{2}and p_{PN}$ with their finite sample estimates.

Supplemental File 4. Analysis of 51 samples that had a non-reactive in-country Architect test and a reactive HPTN LC Architect test.

A. Analysis of samples with discordant site and LC Architect tests

For quality control, approximately 10% of the samples that had a non-reactive in-country test were tested at the HPTN LC with the same assay (Figure 1A); additional samples were also tested at the HPTN LC with the Architect test during evaluation of cases with across-visit HIV status discrepancies. Fifty-one of 10,731 samples tested had discordant Architect test results (non-reactive in-country Architect test; reactive HPTN LC Architect test). Additional testing was performed at the HPTN LC to characterize those samples. Based on this testing, the samples were classified as POS, NEG, POS ACUTE, or INC (Table). Twenty-seven samples were confirmed to be from HIV-uninfected individuals based on testing performed at the HPTN LC. The BioRad and BioPlex tests were both reactive for 12 (44.4%) of the 27 samples. Twenty-three samples were classified as HIV-positive (POS or POS ACUTE); nine of the 23 samples had detectable HIV RNA. For the acute sample and for two of the POS samples, HIV RNA was detected, but was below the limit of quantification (<400 copies/mL). The median viral load for the other five POS sample was 17,060 copies/mL (range: 1,190-106,420). HIV status was not determined for one sample (INC).

| **BioRad** | **BioPlex** | **Geenius** | **HIV RNA** | **Number of samples** | **Within-visit HIV status** |
| --- | --- | --- | --- | --- | --- |
| **R** | **R** | Negative | Not detected | 12 | NEG (N=27) |
| NR | **R** | Negative | Not detected | 6 |  |
| **R** | NR | Negative | Not detected | 5 |  |
| NR | NR | Negative | Not detected | 4 |  |
| **R** | **R** | **Positive** | Not detected | 14 | POS (N=22) |
| **R** | **R** | **Positive** | **Detected** | 8 |  |
| **R** | **R** | Negative | **Detected** | 1 | POS ACUTE (N=1) |
| **R** | **R** | Indeterminate | Not detected | 1 | INC (N=1) |

Abbreviations: R: reactive; NR: non-reactive; NEG: negative; POS: positive; INC: inconclusive; N: number.

B. Comparison of signal-to-cutoff values obtained with the site and LC Architect tests.

To evaluate the reason for the discordant test results, we compared the signal-to-cutoff (S/CO) values obtained for the Architect assay at the in-country laboratory and the HPTN LC (Figure). All 51 samples in this sample set had in-country S/CO values <1.0. For the 23 samples that were confirmed to be from HIV-infected individuals, the median S/CO value obtained at the LC for these samples was 835.76 (range: 46.92-1,306.20). In these cases, the non-reactive in-country Architect test results most likely reflected laboratory errors (e.g., sample mixups, testing errors, or data errors). In contrast, the 27 samples that were confirmed to be from HIV-uninfected individuals had lower HPTN LC S/CO values (mostly <10). In these cases, the discordant Architect test results may have reflected variability in assay performance.


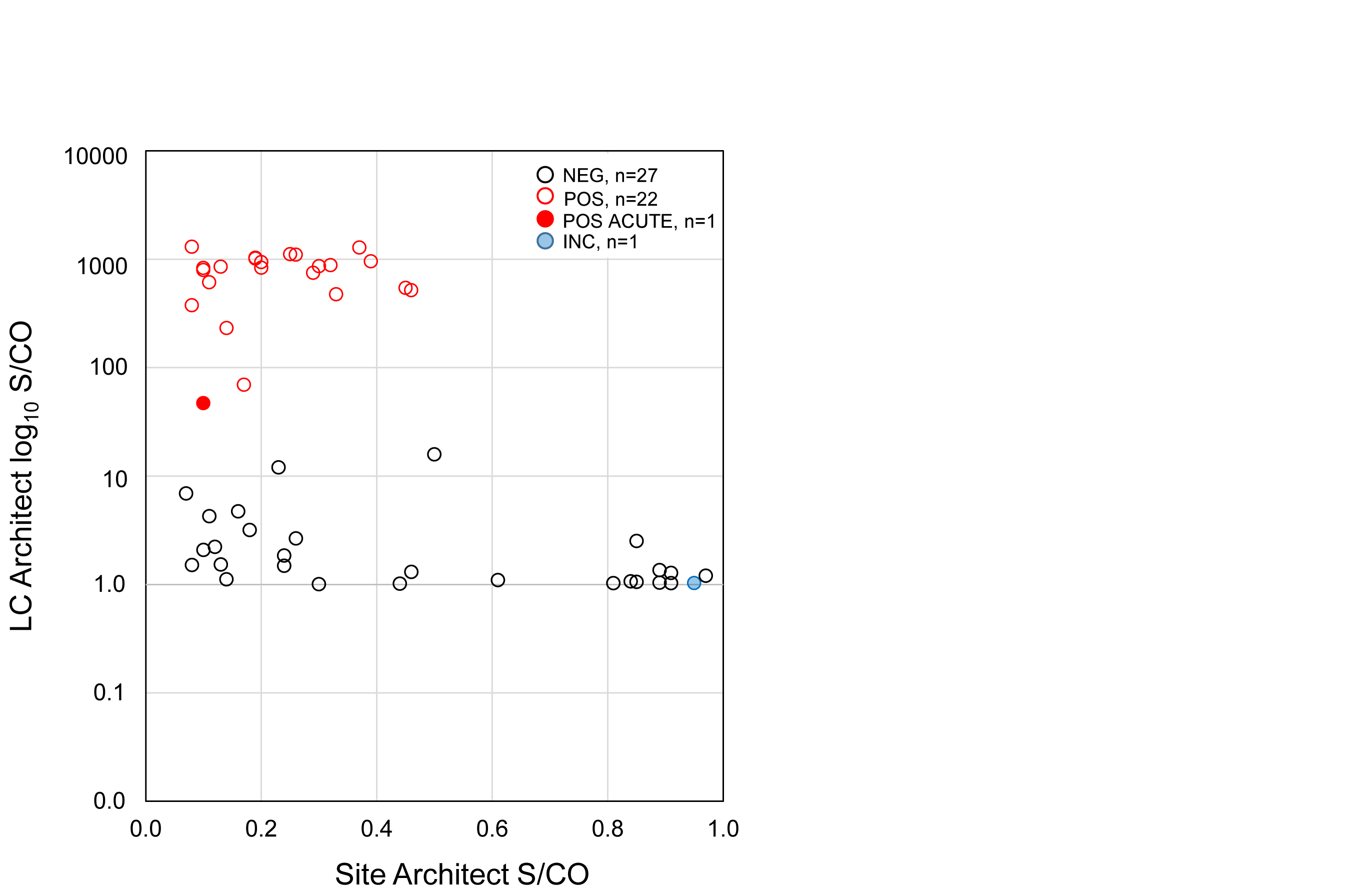


Abbreviations: LC: HPTN Laboratory Center; NEG: within-visit status HIV negative; POS: within-visit status HIV positive; INC: within-visit status HIV inconclusive; n: number.

C. Analysis of discordant samples with triplicate Architect tests.

The Architect test classifies samples with a signal-to-cutoff ratio (S/CO) ≥1.0 as reactive. The manufacturer of this test recommends testing samples in duplicate following an initial S/CO value ≥1.0, and that samples should be classified as reactive only if two of the three S/CO values are ≥1.0. In the HPTN 071 (PopART) trial, the Architect test was performed as a singlet test at the in-country laboratories and at the HPTN LC to streamline testing. As a final step, we evaluated whether concordant in-country site/LC results would have been obtained for the 27 HIV-negative samples if samples with HPTN LC S/CO values ≥1.0 were run in triplicate at the LC, as recommended by the manufacturer. The figure below shows results from the one in-country Architect test, and from three HPTN LC Architect tests.


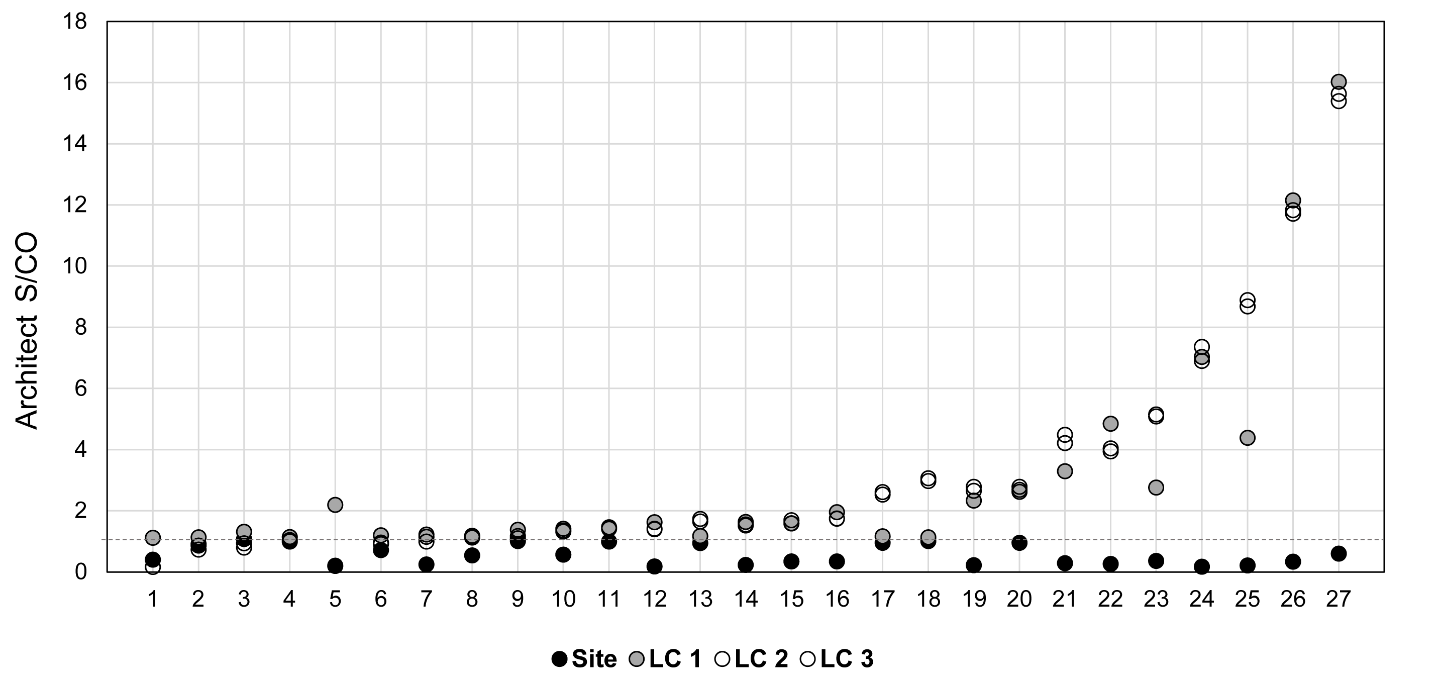


In six (22.2%) of the 27 cases (cases 1-6), the S/CO ratio was near the assay cutoff for the initial HPTN LC test (1.01-2.09) and both of the subsequent LC Architect tests were non-reactive; in these cases, the samples would have been classified as non-reactive at the HPTN LC with triplicate testing. In one case, only one of the two subsequent LC Architect tests was non-reactive; the S/CO ratios were near the assay cut-off for all three LC Architect tests in that case (1.12, 1.05, 0.89). In the 20 remaining cases, the S/CO values for all three of the LC Architect tests were ≥1.0, indicating that triplicate testing would not have resolved the test discrepancy; the S/CO values for these samples with triplicate testing varied from 1.00 to 15.94.

Some variability is inherent in the Architect test when the S/CO is near 1 (repeat testing may yield values slightly above or below this cutoff). In this study, some cases were consistent with assay variability (with both results near the assay cutoff), while others were consistent with sample/data mixups/errors (e.g., one clearly non-reactive, one reactive with a high S/CO ratio). Information was not available to determine whether other factors (e.g., inter-laboratory or inter-technologist variation) contributed to variability in test results. It is notable that the two tests were performed using different sample aliquots, one of which was shipped to the US. It is not possible to determine if this additional handling impacted the test results.

Abbreviations: LC: HPTN Laboratory Center; R: reactive; NR: non-reactive.

Supplemental File 5. Analysis of 227 samples that had a reactive in-country Architect test and a non-reactive HPTN LC BioRad or BioPlex test.

A. Analysis of samples with discordant site Architect tests and HPTN LC BioRad/BioPlex tests.

In HPTN 071 (PopART), 26,842 samples that had a reactive in-country Architect test were also tested at the HPTN LC with the BioRad or BioPlex test (1A); 227 of those samples had discordant test results (reactive in-country Architect test; non-reactive HPTN LC BioRad/BioPlex test). Additional testing was performed at the HPTN LC to characterize those samples. Based on this testing, the samples were classified as NEG, POS, POS ACUTE, or INC (Table). The three acute samples had viral loads of 62,610, 78,630 and 95,340 copies/mL; the other five HIV-positive samples had undetectable HIV RNA.

| **LC**  **Architect** | **BioRad** | **BioPlex** | **Geenius** | **HIV RNA** | **Number of samples** | **Within-visit HIV status** |
| --- | --- | --- | --- | --- | --- | --- |
| NR | NR | NR | Negative | Not detected | 109 | NEG (N=206) |
| **R** | NR | NR | Negative | Not detected | 44 |  |
| NR | NR | **R** | Negative | Not detected | 25 |  |
| **R** | NR | **R** | Negative | Not detected | 15 |  |
| NR | **R** | NR | Negative | Not detected | 4 |  |
| **R** | **R** | NR | Negative | Not detected | 4 |  |
| **R** | N/A | NR | Negative | Not detected | 2 |  |
| N/A | NR | NR | Negative | Not detected | 1 |  |
| NR | N/A | NR | Negative | Not detected | 1 |  |
| NR | NR | N/A | Negative | N/A | 1 |  |
| **R** | NR | **R** | **Positive** | Not detected | 4 | POS (N=5) |
| **R** | NR | NR | **Positive** | Not detected | 1 |  |
| **R** | NR | **R** | Negative | **Detected** | 2 | POS ACUTE |
| **R** | **R** | NR | Negative | **Detected** | 1 | (N=3) |
| **R** | NR | NR | Indeterminate | Not detected | 4 | INC (N=13) |
| **R** | **R** | NR | Indeterminate | Not detected | 4 |  |
| NR | NR | NR | Indeterminate | Not detected | 3 |  |
| NR | NR | **R** | Indeterminate | Not detected | 1 |  |
| **R** | NR | **R** | Indeterminate | Not detected | 1 |  |

Abbreviations: R: reactive; NR: non-reactive; NEG: negative; POS: positive; INC: inconclusive; N: number; N/A: not available (insufficient plasma volume)

B. Comparison of signal-to-cutoff values obtained with the site and LC Architect tests.

To evaluate the reason for the discordant test results, we compared the signal-to-cutoff (S/CO) values obtained for the Architect test at the in-country laboratory and the HPTN LC (Figure; note that BioRad/BioPlex results are not shown on the figure). The graph shows that the S/CO values obtained for the two Architect tests were similar for a subset of the samples that included all eight HIV-positive samples and most of the samples with inconclusive HIV status. Another subset of samples had S/CO values at the in-country lab that were above the assay cut-off (mostly 1-10) and had S/CO values at the HPTN LC that were below the assay-cutoff (<1); those discrepancies may have reflected assay variability. The remaining samples had high S/CO values at the in-country laboratories (e.g., >10) with lower S/CO at the HPTN LC (all <1, non-reactive); in those cases, the discrepant Architect test results (in-country vs. LC) most likely reflected a laboratory error (e.g., sample mixup, testing error, or data error).


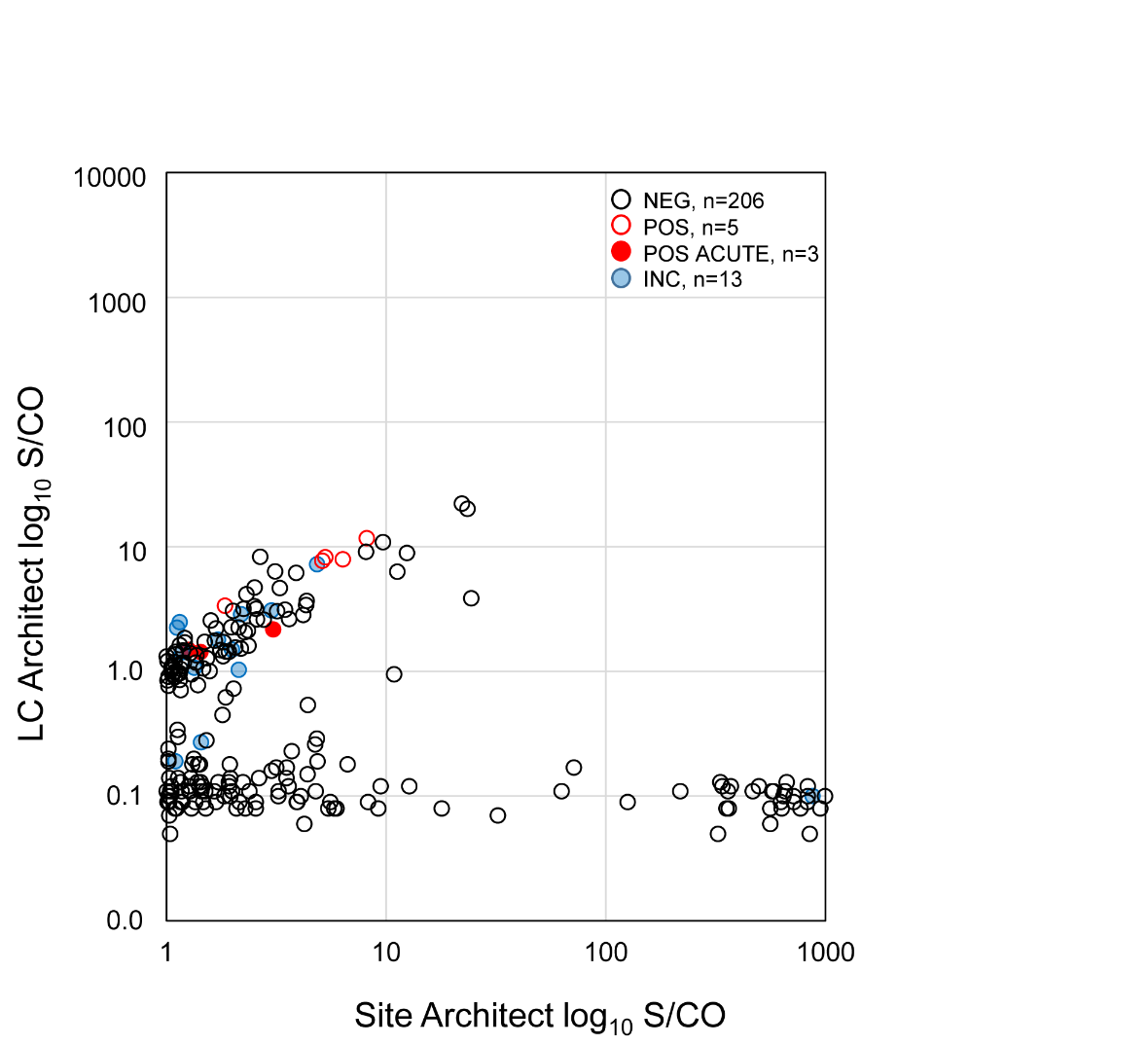


Abbreviations: LC: HPTN Laboratory Center; S/CO: signal-to-cutoff ratio; NEG: within-visit status HIV-uninfected; POS: within-visit status HIV-infected; INC: within-visit status inconclusive; n: number.

Supplemental File 6. Characteristics of acute HIV infections.

Additional testing was performed retrospectively at the LC for the acute infection samples so that the full sample set would have test results from all five tests (Architect test, BioRad test, BioPlex test, HIV RNA test, Geenius test; see Table below). The median HIV viral load of the 28 samples was 98,420 copies/mL (range <400 to 11,231,060). Overall, 17 samples (60.7%) were reactive with the LC Architect test, 17 samples (60.7%) were reactive with the BioRad test, and 22 (78.6%) were reactive with the BioPlex test. This included 14 cases that were reactive with all three screening tests (LC Architect, BioRad, BioPlex), five that were reactive with two of the three screening tests, and four that were reactive with one screening test only (BioPlex). Five cases were non-reactive with all three tests; the viral load in those cases ranged from <400 to 43,360 copies/mL. The 22 cases that were reactive with the BioPlex test included seven that were reactive for antigen only, five that were reactive for antibody only, and 10 that were reactive for both antigen and antibody. The seven samples that were reactive for antigen only had viral loads ranging from 38,150 to 11,321,060 copies/mL; the 11 samples that were non-reactive for antigen had viral loads ranging from <400 to 95,340 copies/mL. Four of the 28 samples had viral loads <400 copies/mL; HIV seroconversion was documented at a subsequent visit in three of these cases (in the other case, acute infection was detected at the last study visit).

| **#** | **Visit** | **Site Architect** | **LC Architect** | **Site S/CO** | **LC S/CO** | **Biorad** | **BioPlex** | **BioPlex Ab** | **BioPlex Ag** | **VL** |
| --- | --- | --- | --- | --- | --- | --- | --- | --- | --- | --- |
| 1 | PC0 | NR | NR | 0.20 | 0.13 | NR | NR | NR | NR | 1,770 |
| 2 | PC12 | NR | NR | 0.32 | 0.10 | NR | NR | NR | NR | <400 |
| 3 | PC12 | NR | NR | 0.12 | 0.09 | NR | NR | NR | NR | 1,050 |
| 4 | PC0 | NR | NR | 0.10 | 0.12 | NR | NR | NR | NR | <400 |
| 5 | PC12 | NR | NR | 0.49 | 0.23 | NR | NR | NR | NR | 43,360 |
| 6 | PC0 | NR | NR | 0.64 | 0.51 | NR | **R** | NR | **R** | 38,150 |
| 7 | PC12 | NR | NR | 0.10 | 0.11 | NR | **R** | **R** | NR | <400 |
| 8 | PC24 | NR | NR | 0.27 | 0.14 | NR | **R** | **R** | NR | 5,210 |
| 9 | PC0 | NR | NR | 0.55 | 0.54 | NR | **R** | **R** | **R** | 47,540 |
| 10 | PC0 | NR | NR | 0.68 | 0.58 | **R** | **R** | **R** | **R** | 13,040 |
| 11 | PC36* | **R** | NR | 1.04 | 0.56 | **R** | **R** | **R** | NR | <400 |
| 12 | PC0 | **R** | **R** | 1.26 | 1.48 | NR | **R** | **R** | NR | 78,630 |
| 13 | PC12 | **R** | **R** | 1.43 | 1.43 | NR | **R** | **R** | NR | 62,610 |
| 14 | PC36* | **R** | **R** | 3.07 | 2.16 | **R** | NR | NR | NR | 95,340 |
| 15 | PC12 | **R** | **R** | 88.58 | 81.19 | **R** | **R** | NR | **R** | 3,911,690 |
| 16 | PC12 | **R** | **R** | 5.50 | 5.37 | **R** | **R** | NR | **R** | 705,290 |
| 17 | PC36* | **R** | **R** | 26.56 | 40.17 | **R** | **R** | NR | **R** | 2,888,650 |
| 18 | PC36* | **R** | **R** | 34.63 | 51.60 | **R** | **R** | NR | **R** | 147,690 |
| 19 | PC12 | **R** | **R** | 8.45 | 11.17 | **R** | **R** | NR | **R** | 409,570 |
| 20 | PC36* | **R** | **R** | 219.90 | 233.63 | **R** | **R** | NR | **R** | 11,231,060 |
| 21 | PC24 | **R** | **R** | 3.20 | 4.18 | **R** | **R** | **R** | **R** | 151,720 |
| 22 | PC12 | **R** | **R** | 12.01 | 16.45 | **R** | **R** | **R** | **R** | 486,430 |
| 23 | PC12 | **R** | **R** | 4.44 | 5.28 | **R** | **R** | **R** | **R** | 140,990 |
| 24 | PC24 | **R** | **R** | 1.41 | 1.04 | **R** | **R** | **R** | **R** | 101,500 |
| 25 | PC36* | **R** | **R** | 28.40 | 24.49 | **R** | **R** | **R** | **R** | 332,720 |
| 26 | PC12 | **R** | **R** | 38.04 | 65.65 | **R** | **R** | **R** | **R** | 208,690 |
| 27 | PC24 | **R** | **R** | 57.53 | 54.06 | **R** | **R** | **R** | **R** | 1,341,980 |
| 28 | PC24 | **R** | **R** | 1.10 | 1.38 | **R** | **R** | **R** | **R** | 69,050 |

Table footnotes.

Twenty-eight acute HIV infections were identified in the study; acute infection was defined as having detectable HIV RNA test with a negative Geenius test. The table shows the results from different HIV assays (see Supplemental Table 1). An asterisk indicates that the acute study visit was the participant’s final visit.

Abbreviations: LC: (HPTN) Laboratory Center; S/CO: signal-to-cutoff ratio; Ag: antigen; Ab: antibody; VL: viral load; R: reactive; NR: non-reactive.

Supplemental File 7. Pattern of HIV test results in seroreverter cases.

Overall, 213 cases were classified as seroreverters, including 154 cases that had a within-visit status of POS at enrollment, and 59 cases that had a within-visit status of NEG at enrollment. The patterns obtained for within-visit status are shown in the table below for the 59 cases with a NEG enrollment status.

| Enr | PC0 | PC12 | PC24 | PC36 | Number of cases |
| --- | --- | --- | --- | --- | --- |
| PC0 | NEG | POS | NEG | NEG | 13 |
| PC0 | NEG | POS | NEG | POS | 4 |
| PC0 | NEG | POS | NEG | MISSING | 3 |
| PC0 | NEG | POS | MISSING | NEG | 4 |
| PC0 | NEG | MISSING | POS | NEG | 2 |
| PC0 | NEG | NEG | POS | NEG | 23 |
| PC12 |  | NEG | POS | NEG | 10 |

Abbreviations: Enr: enrollment; NEG: within-visit status HIV uninfected; POS: within-visit status HIV infected.

In HPTN 071, seroreversion cases represented 0.64% of the total cases where participants had HIV status determined at two or more visits. It would not be surprising to have this frequency of sample/data errors, particularly in a study of this size where sample collection was performed in homes and where sample processing and aliquot labeling were performed in multiple laboratories because of the geographic distances involved.

Other studies have found unusual HIV test patterns in some individuals (e.g., individuals with co-receptor defects and those infected with defective viruses). It is unlikely that these factors were responsible for the seroreversion events in HPTN 071, based on the type of HIV test results obtained in these cases. In this study, all seroreverter cases had a visit with multiple reactive/positive assays including a positive Geenius test, followed by a visit where all assays were non-reactive/negative. None of these cases had inconclusive test results at the HIV POS or subsequent HIV NEG visit. Also, there were no cases where participants had more than one “RNA only” visit (i.e., cases with intermittent and/or transient viremia). In all cases where the participant had an RNA-only visit with a subsequent follow-up visit, the acute infection visit was followed by a visit with a positive Geenius test. Of note, it was not possible to perform host genetic testing in this study because this type of testing was not included in study consents.
